# CircRBM33 downregulation inhibits hypoxia-induced glycolysis and promotes apoptosis of breast cancer cells via a microRNA-542-3p/HIF-1α axis

**DOI:** 10.1038/s41420-022-00860-6

**Published:** 2022-03-22

**Authors:** Yiming Jiang, Meiqi Zhang, Danlu Yu, Guoxin Hou, Jingyi Wu, Fuming Li

**Affiliations:** 1 Department of Oncology, Affiliated Hospital of Jiaxing University, Jiaxing, 314000 Zhejiang Province China; 2 Department of Outpatient, Affiliated Hospital of Jiaxing University, Jiaxing, 314001 Zhejiang Province China; 3 Department of Endocrinology, Affiliated Hospital of Jiaxing University, Jiaxing, 314001 Zhejiang Province China; 4 Department of Breast Surgery, Second Hospital of Jiaxing City, Jiaxing, 314000 Zhejiang Province China

**Keywords:** Breast cancer, Cancer immunotherapy

## Abstract

Many circRNAs are involved in the carcinogenesis of breast cancer (BCa) through the transcription of microRNAs (miRNAs) and mRNAs. This study investigated circRBM33 regulation of the miR-542-3p/hypoxia-inducible factor-1α (HIF-1α) axis in BCa. BCa clinical tissue samples were collected to test differential expressions of circRBM33, miR-542-3p, and HIF-1α. MCF-7 cells were subjected to normoxia or hypoxia and transfected with plasmids that regulated CircRBM33, miR-542-3p, and HIF-1α expression levels. Glycolysis was evaluated by measuring glucose consumption, lactic acid production, and protein expression of hexokinase 2, glucose transporter type 1 and lactic dehydrogenase A. Cell proliferation and apoptosis were also assessed, and the interactions between genes were explored. CircRBM33 and HIF-1α were upregulated, while miR-542-3p was downregulated in BCa tissue samples and cell lines. Hypoxia induced circRBM33 expression in BCa cells, which negatively regulated miR-542-3p expression. CircRBM33 knockdown or miR-542-3p rescue reduced glycolysis and proliferation and promoted apoptosis of BCa cells. MiR-542-3p inhibition rescued circRBM33 knockdown-mediated glycolysis, proliferation and apoptosis of BCa cells. MiR-542-3p targeted HIF-1α, and the overexpression of HIF-1α reversed the effect of miR-542-3p upregulation on glycolysis, proliferation, and apoptosis of BCa cells. Collectively, downregulating circRBM33 suppresses miR-542-3p-targeted HIF-1α expression, resulting in the inhibition of glycolysis and proliferation and the promotion of BCa cells’ apoptosis.

## Introduction

Breast cancer (BCa) is the fifth leading cause of death [1] and manifests with symptoms including redness or rash around the skin or nipples, changes in breast size or shape, lumps or thickening, nipple discharge, axilla swelling, and persistent pain [2]. The length of the menstrual life, especially the part that occurs before the first full-term pregnancy, is the main risk for BCa [3]. Primary prevention to eliminate pathogenic factors and to enhance immune system, such as improving diet and reducing alcohol intake, and secondary prevention to detect BCa, such as mammography and ultrasonography, are recommended to the risk of BCa occurrence and development [4]. Tumor cells mainly rely on glycolysis to produce energy, and aerobic glycolysis is a scientifically recognized marker of cancer cell metabolism [5]. Therefore, a targeted regulation of glycolysis may provide a therapeutic direction for cancer treatment.

Oncogenic or anti-oncogenic circRNAs suggest their potential use in the treatment of BCa [6]. For instance, circRNF20 enhances the proliferation and glycolysis of BCa cells [7], while the downregulation of neoplasia domain containing 4 C (circDENND4C) decreases glycolysis and invasiveness of BCa cells under hypoxia [8]. CircRBM33 participates in the progression of gastric cancer (GC), and its expression closely correlated with metastasis, differentiation, position, and size of tumors [9]. It was shown that CircRBM33 is a trigger for glycolysis, and contributes to the growth of cervical cancer (CC) cells [10]; however, research on the mechanism of CircRBM33 in BCa is very limited. MiR-542-3p is an effective target that suppresses BCa resistance to paclitaxel [11], and that has synergistic effects when combined with doxorubicin on fighting [12]. More importantly, miR-542-3p is an indicator of cancer stages and has an antiangiogenic effect on primary BCa [13]. Regarding BCa cellular growth, miR-542-3p suppresses the biological behaviors of BCa cells through regulating its target [14]. Hypoxia provides energy for cancer development, and hypoxia-inducible factor (HIF) regulates many genes related to cancer development [15]. HIF-1α was selected as a target of miR-542-3p via bioinformatics analysis and has been implied to partly orchestrate hypoxia-induced metabolic changes to glycolysis and autophagy of BCa cells [16]. Moreover, Jinmei Jin *et al*. showed that the repression of the HIF-1α-dependent metabolism blocks glycolysis and growth of BCa cells [17]. CircRNAs roles as mediators of microRNAs (miRNAs) transcriptional regulatory functions associated with the development of cancer are widely accepted. Based on this background, we hypothesized that the circRBM33/miR-542-3p/HIF-1α axis may mediate BCa growth and progression. This mechanism may involve a role of circRBM33 in enhancing the growth and glycolysis of BCa cells through upregulating miR-542-3p targeting of HIF-1α.

## Methods and materials

### Clinical samples

BCa and normal tissue specimens were excised from 56 patients (>18 years old) in Second Hospital of Jiaxing City from September 2016 to March 2018. All cases were diagnosed as BCa and received no neoadjuvant radiotherapy or chemotherapy before surgery.

### Culture and transfection of cells

The human normal breast epithelial cell line, MCF-10A, and the human BCa cell lines, MCF-7, MDA-MB-231, and SKBR3A (ATCC, VA, USA), were cultured in Roswell Park Memorial Institute-1640 medium with 10% fetal bovine serum (FBS). For hypoxic induction, the cells were exposed to 1% O_2_, 5% CO_2_, and 94% N_2_.

After reaching a 70–80% confluence, the cells were detached using 0.25% trypsin and passaged. The cells were divided into a normoxia group (without hypoxic induction) and an hypoxia group (hypoxic induction for 24 h) and transfected with si-negative control (NC), si-CircRBM33, miR-NC, miR-542-3p/miR-149/miR-758-3p inhibitor, miR-542-3p/miR-149/miR-758-3p mimic, overexpressed (oe)-NC, and oe-HIF-1α, respectively, using Li-pofectamine 2000.

### Reverse transcription quantitative polymerase chain reaction (RT-qPCR)

Total RNA in tissues and cells was extracted by Trizol method (Takara, Dalian, China) and analyzed to determine concentration and purity. The reverse transcription of RNA into cDNA was conducted using the PrimeScript RT kit (Takara). The fluorescent quantitative PCR was processed on the ABI PRISM® 7300 system (Applied Biosystems, MA, USA). Taking Glyceraldehyde-3-phosphate dehydrogenase (GAPDH) was used as an internal control, and gene expression was measured using the 2^−△△Ct^ method [18]. The primer sequences (Table 1) were designed by Sangon (Shanghai, China).

**Table 1:** Primer sequences.

Name of primers	Sequences
miR-542-3p-F	TCGGGGATCATCATGTCACG
miR-542-3p-R	GAGTGGCTCCCAGACCTTTC
U6-F	TCGCTTCGGCAGCACATATAC
U6-R	GCGTGTCATCCTTGCGCAG
CircRBM33-F	CCCAGAAGAAGGACAGTATGAA
CircRBM33-R	TGTAACACCCTGAGAACTGAAAT
HIF-1α-F	GAAAGCGCAAGTCTTCAAAG
HIF-1α-R	TGGGTAGGAGATGGAGATGC
GAPDH-F	ACCACAGTCCATGCCATCAC
GAPDH-R	TCCACCACCCTGTTGCTGTA

Note: *F* forward; *R* reverse; *miR-542-3p* microRNA-542-3p; *CircRBM33* Circular RNA RBM33; *HIF-1α* Hypoxia-inducible factor-1α; *GAPDH* glyceraldehyde-3-phosphate dehydrogenase.

### Western blot assay

Protein samples were obtained from cells after treatment with radio-immunoprecipitation assay lysis solution (Beyotime, Shanghai, China) and then quantified by the bicinchoninic acid kit (Beyotime). The proteins were mixed with the sample buffer (Beyotime), denatured, and electrophoresed at 80 V. After transferring onto a membrane, this latter was blocked in the blocking solution, incubated with the primary antibodies, HIF-1α (1:6000, ab205718), hexokinase 2 (HK2, 1:1000, ab209847), LDHA (1:5000, ab52488), GLUT1 (1:100000, ab115730, (Abcam, MA, USA), and then washed in PBS and incubated with secondary horseradish peroxidase-labeled goat anti-rabbit immunoglobulin G (IgG) antibody (1:5000, CWBIO, Beijing, China). The membrane was developed, and protein bands were visualized using a chemiluminescence imaging system (Bio-rad, CA, USA).

### Detection of glucose consumption and lactate production

The supernatant of each cell culture medium was collected to determine glucose and lactic acid concentrations with the glucose colorimetric determination kit and lactic acid determination kit (both from Biovision, Wehrheim, Germany). The relative concentration was normalized to total proteins [19].

### Cell counting kit (CCK)−8 assay

The cells were trypsinized, stained with trypan blue staining, supplemented with Dulbecco’s Modified Eagle Medium and FBS, and added to a 96-well plate with 2000 cells/well for 24, 48, 72 h, respectively. After culture, the cells were incubated with CCK-8 solution (10 μL/well) for 3 h, and optical density at 450 nm was measured on a microplate reader.

### Flow cytometry

Annexin V-fluorescein isothiocyanate (FITC) Apoptosis Detection Kit (Thermo Fisher Scientific, MA, USA) was used to measure cell apoptosis. Cell suspension (1 × 10^6^ cells/mL, 200 µL) was mixed with binding buffer (300 µL), supplemented with Annexin V-FITC/propidium iodide (5 µL) and detected on a flow cytometer.

### Dual luciferase reporter gene assay

The binding sites of circRBM33 and miR-542-3p and that of miR-542-3p and HIF-1α were predicted online using http://starbase.sysu.edu.cn/. Wild-types circRBM33 (WT-CircRBM33) and HIF-1α (WT-HIF-1α) reporters were constructed by cloning the sequence of circRBM33 or HIF-1α containing miR-542-3p complementary sequence into the pmirGLO vector (Promega, WI, USA). The site-directed mutant reporters MUT-CircRBM33 and MUT-HIF-1α were produced by site-directed mutagenesis kit (Yeasen, Shanghai, China). The reporter, along with miR-542-3p mimic or miR-NC, were co-transfected into cells to detect luciferase activity on the dual luciferase reporter gene detection system (Promega).

### RNA immunoprecipitation (RIP) assay

RIP analysis was performed with Magna RIP kit (Millipore, MA, USA). Magnetic beads coated with human anti-Ago2 antibody were reacted with RIP buffer and cell lysates. Normal IgG was used as a negative control (NC). After treatment with proteinase K, RNA was used for RT-qPCR.

### Statistical analysis

The statistical analysis was performed using GraphPad prism7, and all data were expressed as mean ± standard deviation. The comparison between two groups was performed by *t* test, multiple groups by one-way analysis of variance (ANOVA), while pairwise comparison was performed by the Tukey’s method. The correlation was assessed using Pearson correlation analysis. *P* < 0.05 was indicative of a statistically significant difference.

## Results

### CircRBM33 is upregulated in BCa tissues and hypoxia-induced BCa cells

**Fig. 1:**
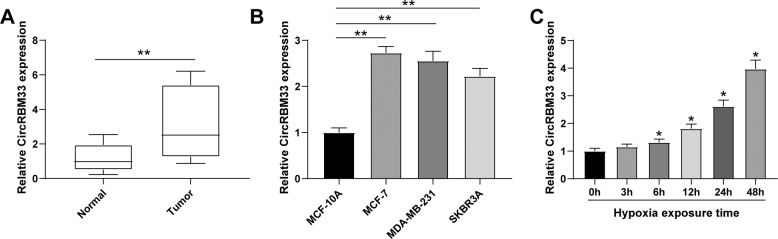
CircRBM33 is upregulated in BCa tissues and hypoxia-induced BCa cells. **A** CircRBM33 was upregulated in BCa tissues; **B** CircRBM33 was upregulated in BCa cells (Compared with MCF-10A cells, circRBM33 expression was 1.7 times higher in MCF-7 cells); **C** CircRBM33 was upregulated in hypoxia-induced MCF-7 cells; **P < 0.01; *P < 0.05 compared with the 0 h group. Data were expressed in the form of mean ± standard deviation (Repetitions = 3). The comparison between two groups was performed by *t* test, that among multiple groups was by one-way ANOVA, and pairwise comparison was by Tukey’s method.

To explore the potential role of circRBM33 in BCa, we measured its expression in BCa tissues and cell lines using RT-qPCR and found that circRBM33 is highly expressed in BCa tissue samples compared with that of normal tissue samples (Fig. 1A). CircRBM33 expression was also higher in human BCa cell lines (MCF-7, MDA-MB-231, and SKBR3A) compared with that in the MCF-10A normal breast epithelial cells (Fig. 1B). Since the highest level of circRBM33 was seen in MCF-7 cells, MCF-7 cells were selected for subsequent experiments. After hypoxia induction, circRBM33 expression increased in MCF-7 cells in a time-dependent manner (Fig. 1C). These results demonstrate that circRBM33 is highly expressed in BCa, and that its expression is induced under hypoxic conditions.

### Hypoxia increases glycolysis and inhibits apoptosis of BCa cells

**Fig. 2:**
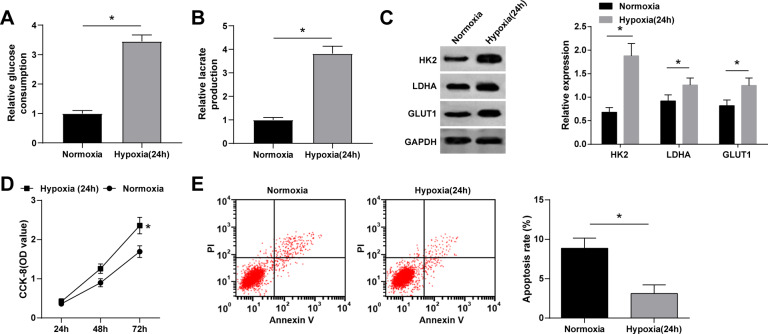
Hypoxia increases glycolysis and inhibits apoptosis of MCF-7 cells. **A** Glucose consumption of MCF-7 cells under hypoxic conditions was 2.5 times that under normoxia; **B** Lactate production of MCF-7 cells under hypoxic conditions was 2.8 times that under normoxia; **C** Western blot detected HK2, LDHA and GLUT1 expression under hypoxia (HK2 expression of MCF-7 cells under hypoxic conditions was 2.8 times that under normoxia, LDHA expression of MCF-7 cells under hypoxic conditions was 1.4 times that under normoxia, GLUT1 expression of MCF-7 cells under hypoxic conditions was 1.9 times that under normoxia); **D** CCK-8 detected MCF-7 cell proliferation under hypoxia; **E** Flow cytometry detected MCF-7 cell apoptosis under hypoxia (cell apoptosis rate decreased from 8.86% to 3.12%); *P < 0.05. Data were expressed in the form of mean ± standard deviation (Repetitions = 3). The comparison between two groups should be performed by *t* test.

Several studies reported that hypoxia promotes glycolysis and proliferation and inhibits apoptosis of cancer cells [20–24]. Therefore, glycolysis and apoptosis of BCa cells were tested under normoxia and hypoxia. The results showed an increased cell glucose consumption and lactate production under hypoxic conditions compared with those normoxic conditions (Fig. 2A, B). The protein expression levels of the glycolysis-related proteins, HK2, LDHA, and GLUT1, were detected by Western blot, and the results showed that HK2, LDHA, GLUT1, and GLUT1 protein expressions increased under hypoxic conditions (Fig. 2C). Cell proliferation and apoptosis were detected by CCK-8 and flow cytometry, respectively. Under hypoxic conditions, cell proliferation was promoted, and cell apoptosis was inhibited (Fig. 2D, E) (all *P* < 0.05). In summary, glycolysis, and cell proliferation of BCa cells can be induced under hypoxic conditions, while apoptosis is inhibited.

### Knockout of circRBM33 reduces glycolysis and promotes apoptosis of BCa cells

**Fig. 3:**
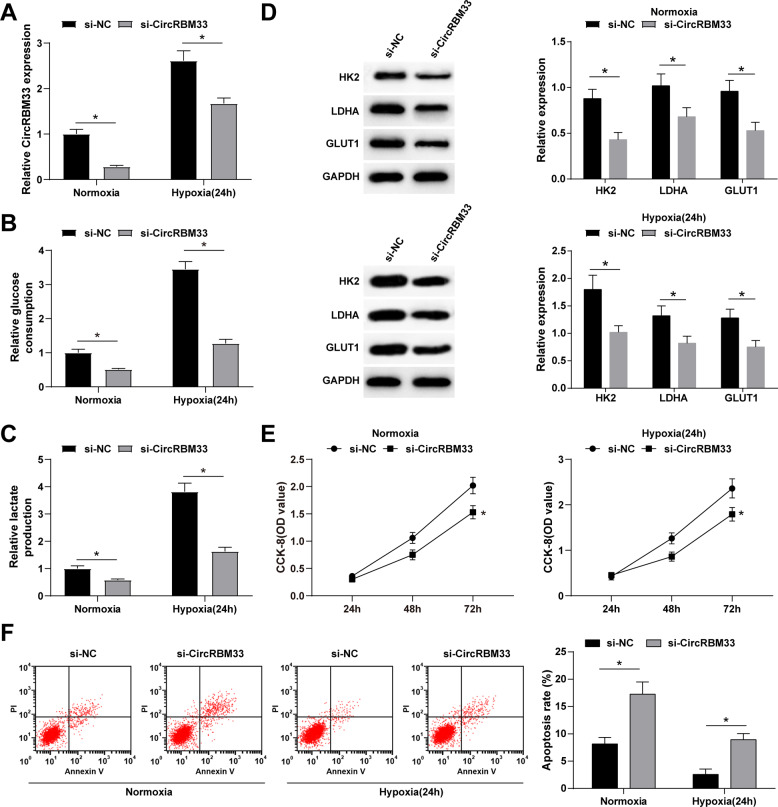
Knockout of circRBM33 reduces glycolysis and promotes apoptosis of BCa cells. **A** CircRBM33 expression in MCF-7 cells after knocking down circRBM33; **B** Glucose consumption in MCF-7 cells after knocking down circRBM33; **C** Lactate production in MCF-7 cells after knocking down circRBM33; **D** Western blot detected HK2, LDHA and GLUT1 expression in MCF-7 cells after knocking down circRBM33; **E** CCK-8 detected MCF-7 cell proliferation after knocking down circRBM33; **F** Flow cytometry detected MCF-7 cell apoptosis after knocking down circRBM33 (Under normoxia, the apoptotic rate increased from 8.15% to 17.25% after knocking down circRBM33; Under hypoxia, the apoptotic rate increased from 2.58% to 8.95% after knocking down circRBM33); *P < 0.05. Data were expressed in the form of mean ± standard deviation (Repetitions = 3). The comparison between two groups should be performed by *t* test.

It was previously reported that circRBM33 knockdown inhibits cervical cancer glycolysis [25]. CircRBM33 is overexpressed in BCa and can be induced under hypoxic conditions. To further study the mechanism by which hypoxia induces glycolysis and cell proliferation and inhibits apoptosis of BCa cells, we designed si-CircRBM33 to knock down CircRBM33 expression in BCa cells (Fig. 3A). Transfection of si-CircRBM33 reduced glucose consumption and lactate production of BCa cells in normoxia and hypoxia groups (Fig. 3B, C). Western blot analysis showed that circRBM33 downregulation decreases HK2, LDHA, GLUT1 and GLUT1 protein expressions in both normoxic and hypoxic BCa cells (Fig. 3D). The results of CCK-8 and flow cytometry demonstrated that si-CircRBM33 reduces proliferation and promotes apoptosis of BCa cells in the normoxia and hypoxia groups (Fig. 3E, F). Collectively, these results demonstrate that of circRBM33 knockout reduces glycolysis and proliferation and promotes apoptosis of BCa cells.

### CircRBM33 negatively regulates miR-542-3p expression

**Fig. 4:**
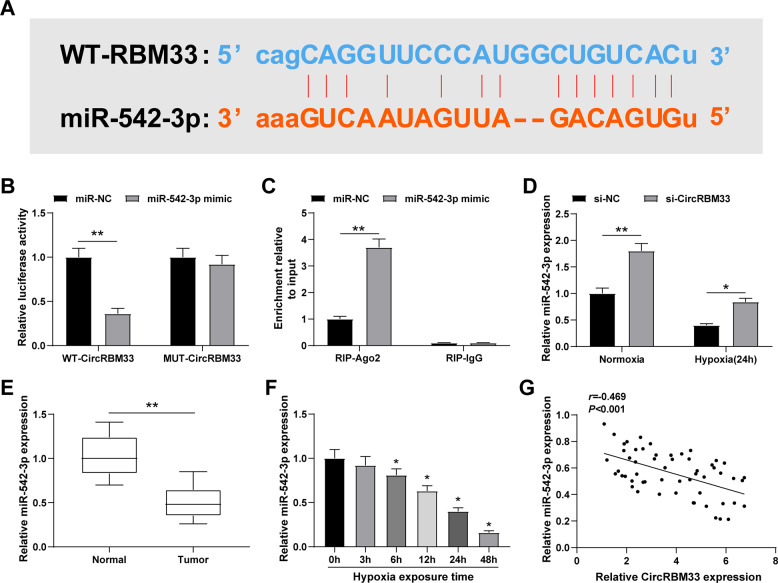
CircRBM33 negatively regulates miR-542-3p expression. **A** StarBase database showed binding sites between circRBM33 and miR-542-3p; **B** Dual luciferase reporter experiment confirmed the interaction between circRBM33 and miR-542-3p; **C** RIP experiment confirmed the interaction between circRBM33 and miR-542-3p; **D** MiR-542-3p expression after knocking down circRBM33; **E** miR-542-3p was downregulated in BCa tissues; **F** MiR-542-3p was downregulated in hypoxia-induced BCa cells; G. Pearson test analyzed the correlation between circRBM33 and miR-542-3p levels; **P < 0.01; *P < 0.05. Data were expressed in the form of mean ± standard deviation (Repetitions = 3). The comparison between two groups should be performed by *t* test, that among multiple groups was by one-way ANOVA, while pairwise comparison was by Tukey’s method. The correlation was analyzed using Pearson correlation analysis.

The StarBase database showed that circRBM33 and miR-542-3p may bind to each other (Fig. 4A). The interaction between the two was confirmed by dual luciferase reporter experiment, as shown by the reduction of the luciferase activity of the cells that were co-transfected with miR-542-3p mimic and WT-CircRBM33 (Fig. 4B). To verify the endogenous association between circRBM33 and miR-542-3p, RIP experiment was conducted in MCF-7 cells overexpressing miR-542-3p using an anti-Ago2 antibody. The results demonstrated that miR-542-3p overexpression enhances circRBM33 enrichment (Fig. 4C).

Furthermore, miR-542-3p expression was measured by RT-qPCR, and the finding indicated that miR-542-3p was increased when circRBM33 was inhibited in the cells (Fig. 4D). Moreover, the reduction of miR-542-3p expression was tested in BCa and normal tissue samples (Fig. 4E). We found that miR-542-3p expression is reduced by hypoxia in BCa cells in a time-dependent manner (Fig. 4F). Besides, Pearson test confirmed the negative correlation between circRBM33 and miR-542-3p (Fig. 4G), further indicating that circRBM33 interacts with miR-542-3p in BCa. The above results indicate that circRBM33 negatively regulates miR-542-3p expression through of its interaction with miR-542-3p.

### Inhibition of miR-542-3p rescues circRBM33 knockout-mediated glycolysis and apoptosis for BCa cells

**Fig. 5:**
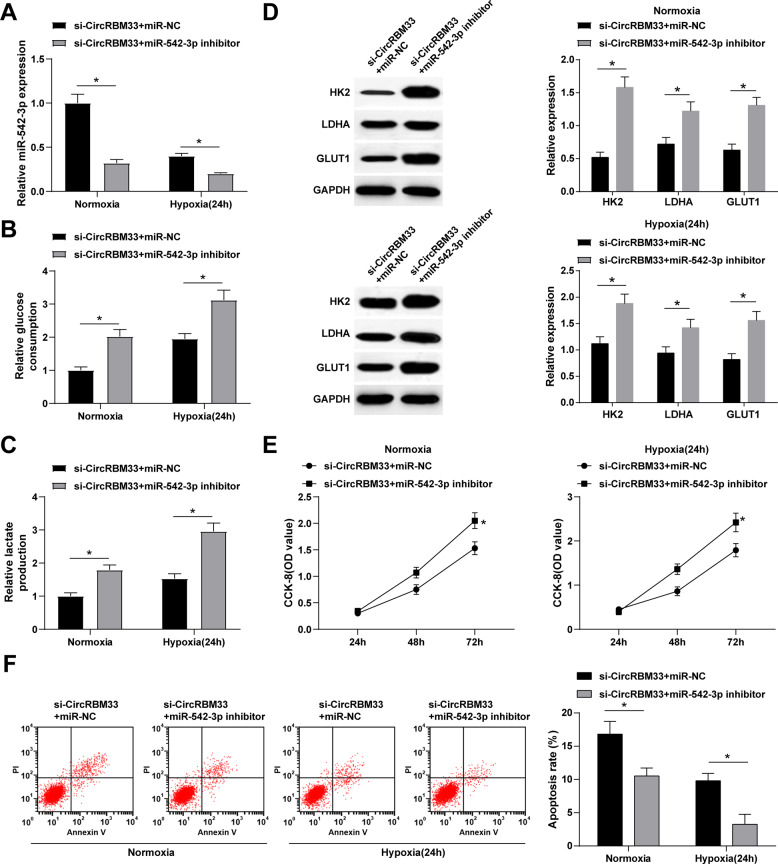
Inhibition of miR-542-3p rescues circRBM33 knockout-mediated glycolysis and apoptosis for BCa cells. **A** miR-542-3p expression in MCF-7 cells after inhibiting circRBM33 and miR-542-3p; **B** Glucose consumption in MCF-7 cells after inhibiting circRBM33 and miR-542-3p; **C** Lactate production in MCF-7 cells after inhibiting circRBM33 and miR-542-3p; **D** Western blot detected HK2, LDHA and GLUT1 expression in MCF-7 cells after inhibiting circRBM33 and miR-542-3p; **E** CCK-8 detected MCF-7 cell proliferation after inhibiting circRBM33 and miR-542-3p; **F** Flow cytometry detected MCF-7 cell apoptosis after inhibiting circRBM33 and miR-542-3p (Under normoxia, the apoptotic rate dropped from 16.82% to 10.52% after inhibiting circRBM33 and miR-542-3p; Under hypoxia, the apoptotic rate decreased from 9.80% to 3.25% after inhibiting circRBM33 and miR-542-3p); *P < 0.05. Data were expressed in the form of mean ± standard deviation (Repetitions = 3). The comparison between two groups should be performed by *t* test.

Subsequently, using a miR-542-3p inhibitor, we successfully inhibited miR-542-3p expression in si-CircRBM33-transfected BCa cells (Fig. 5A). The results showed that the co-transfection of si-CircRBM33 and miR-542-3p inhibitor promotes glucose consumption and lactate production in BCa cells in the normoxia and hypoxia groups (Fig. 5B, C), and increases HK2, LDHA, and GLUT1 protein expressions (Fig. 5D). Cell proliferation and apoptosis analysis revealed that the downregulation of circRBM33 and miR-542-3p accelerates proliferation, while also suppressing apoptosis of BCa cells in the normoxia and hypoxia groups (Fig. 5E, F). In summary, miR-542-3p downregulation can reverse the effect of circRBM33 knockdown on glycolysis, proliferation, and apoptosis of BCa cells.

### MiR-542-3p targets HIF-1α

**Fig. 6:**
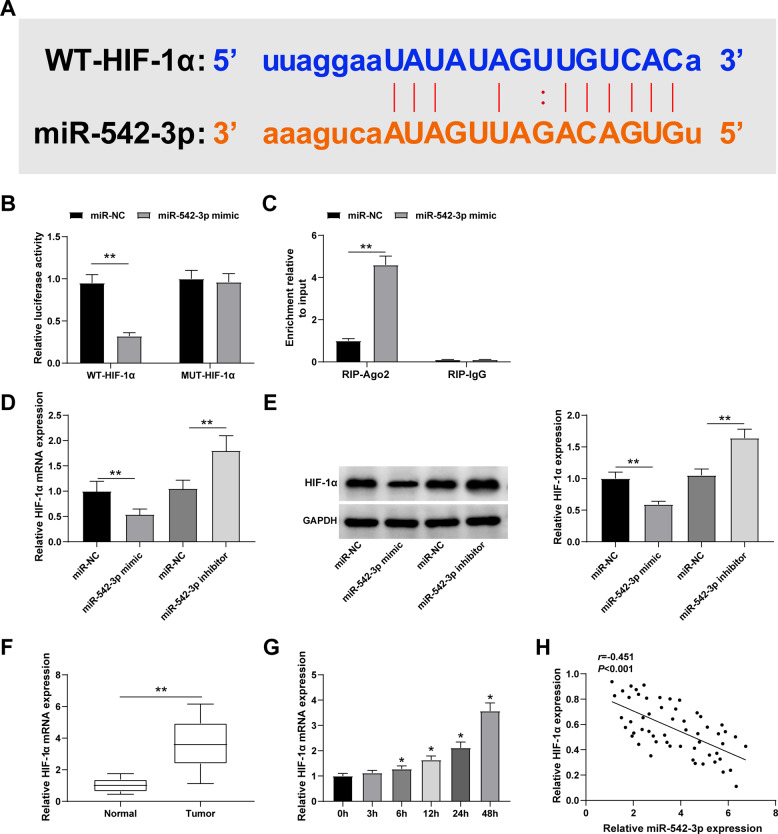
MiR-542-3p targets HIF-1α. **A** The binding site between miR-542-3p and HIF-1α; **B** Dual luciferase reporter experiment confirmed the interaction between miR-542-3p and HIF-1α; **C** RIP experiment confirmed the interaction between miR-542-3p and HIF-1α; **D**, **E** HIF-1α expression after downregulating or upregulating miR-542-3p; **F** HIF-1α was upregulated in BCa tissues; **G** HIF-1α was upregulated in hypoxia-induced BCa cells; **H** Pearson test analyzed the correlation between miR-542-3p and HIF-1α; **P < 0.01; *P < 0.05. Data were expressed in the form of mean ± standard deviation (Repetitions = 3). The comparison between two groups should be performed by *t* test, that among multiple groups was by one-way ANOVA, while pairwise comparison was by Tukey’s method. The correlation was analyzed using Pearson correlation analysis.

Using the StarBase data, we searched for the binding sites between HIF-1α and miR-542-3p (Fig. 6A). The outcome from the dual luciferase reporter experiment showed that the transfection of miR-542-3p mimic weakens the luciferase activity of WT-HIF-1α (Fig. 6B). Meanwhile, RIP detection demonstrated that HIF-1α and miR-542-3p are enriched in Ago2 immunoprecipitation (Fig. 6C).

Subsequently, we applied RT-qPCR and Western blot methods to measure HIF-1α levels in cells and found that HIF-1α expression decreases in the cells transfected with miR-542-3p mimic, while this expression was increased in the cells transfected with the miR-542-3p inhibitor (Fig. 6D, E).

In addition, higher HIF-1α levels were observed in BCa tissue samples compared to those in normal tissue samples, and in hypoxic BCa cells compared to those in normoxic BCa cells (Fig. 6F, G). Pearson analysis confirmed a negative correlation between HIF-1α and miR-542-3p expressions (Fig. 6H). In summary, HIF-1α is a target of miR-542-3p.

### Overexpression of HIF-1α reverses the inhibitory effect of upregulated miR-542-3p on glycolysis and apoptosis of BCa cells

**Fig. 7:**
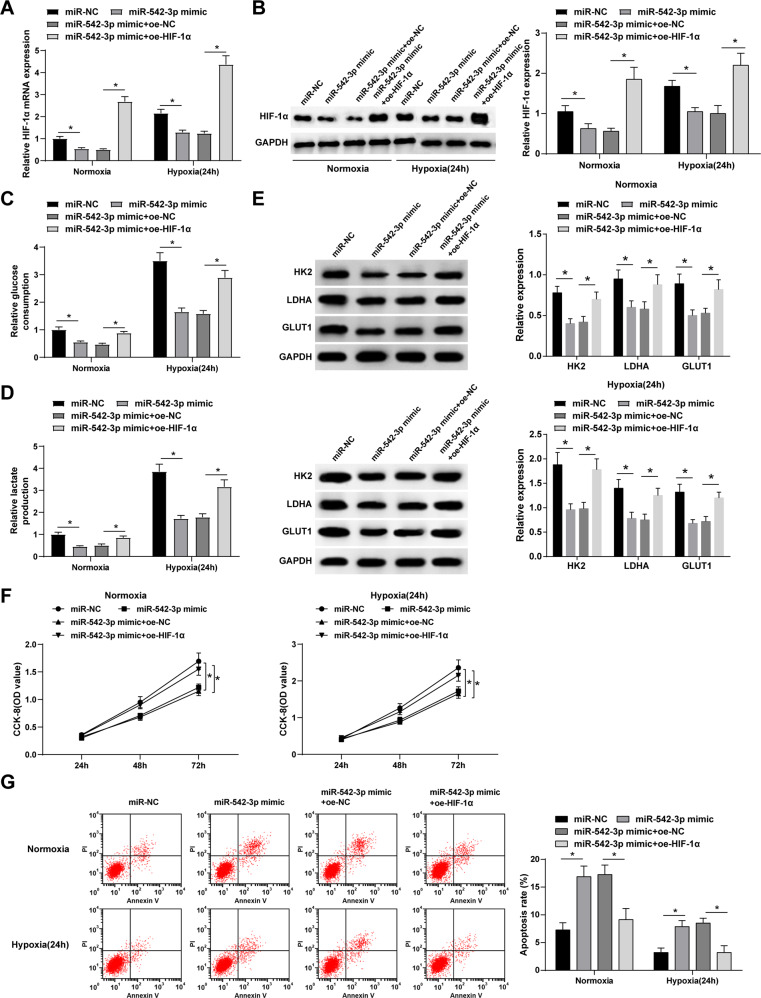
Overexpression of HIF-1α reverses the inhibitory effect of upregulated miR-542-3p on glycolysis and apoptosis of BCa cells. **A**, **B** HIF-1α expression in MCF-7 cells after upregulating miR-542-3p and HIF-1α; **C** Glucose consumption in MCF-7 cells after upregulating miR-542-3p and HIF-1α; **D** Lactate production in MCF-7 cells after upregulating miR-542-3p and HIF-1α; **E** Western blot detected HK2, LDHA and GLUT1 expression in MCF-7 cells after upregulating miR-542-3p and HIF-1α; **F** CCK-8 detected MCF-7 cell proliferation after upregulating miR-542-3p and HIF-1α; **G** Flow cytometry detected MCF-7 cell apoptosis after upregulating miR-542-3p and HIF-1α (Under normoxia, after upregulating miR-542-3p, the apoptotic rate increased from 7.28% to 16.88%, and after upregulating miR-542-3p and HIF-1α, the apoptotic rate decreased from 17.25% to 9.14%; Under hypoxia, after upregulating miR-542-3p, the apoptotic rate increased from 3.20% to 7.88%, and after upregulating miR-542-3p and HIF-1α, the apoptotic rate decreased from 8.50% to 3.340%); *P < 0.05. Data were expressed in the form of mean ± standard deviation (Repetitions = 3). The comparison among multiple groups should be performed by one-way ANOVA, while pairwise comparison was by Tukey’s method.

To further investigate this mechanism, we overexpressed miR-542-3p and HIF-1α in BCa cells (Fig. 7A-B). We found that miR-542-3p mimic inhibits glycolysis and proliferation of BCa cells in the normoxia and hypoxia groups, while the co-transfection of HIF-1α reverses the effects of miR-542-3p mimic on glycolysis and proliferation (Fig. 7C–F). According to the flow cytometry results, we found that the overexpression of miR-542-3p promotes the apoptosis of BCa cells in the normoxia and hypoxia groups, and that such effects can be mitigated when HIF-1α was overexpressed (Fig. 7G). Together, these results demonstrate that miR-542-3p inhibits glycolysis and proliferation and promotes cell apoptosis of BCa cells through HIF-1α.

## Discussion

BCa is a heterogeneous disease that leads to a high mortality rate in female [26]. Our study investigated the mechanism and effects of circRBM33 in BCa. Our results showed that circRBM33 downregulation increases miR-542-3p expression, which reduces HIF-1α expression leading to the suppression of glycolysis and proliferation of BCa cells.

In this study, we first showed that circRBM33 expression is high in BCa tissues, and that its expression is induced by hypoxia. Subsequently, we functionally observed that circRBM33 silencing in BCa cells, under normoxia or hypoxia, reduces glycolysis and proliferation, and enhanced apoptosis of BCa cells. To our best knowledge, other studies reported a similar effect of circRBM33 in other cancer types. It has been shown that circRBM33 silencing inhibits proliferation, migration, and invasion, and promotes apoptosis of gastric cancer cells through targeting the miR-149/IL-6 axis [9]. Furthermore, circRBM33 knockdown have been shown to regulate the miR-758-3p/PUM2 axis to inhibit tumor growth in vivo, suppress cervical cancer cell proliferation, migration, invasion, and glycolysis, and promote cervical cancer cell apoptosis in vitro [25]. However, we found that miR-149 and miR-758-3p do not affect the expression of HIF1α (Supplementary Fig. 1). As indicated, circRBM33 gene expression is upregulated in GC, and the reduction of CircRBM33 expression enhances apoptotic but blocks proliferative, invasive, and migratory phenotypes of GC cells via a targeted increase of miR-149 expression [9]. Another report also reported an increase of circRBM33 expression in CC, and showed that of circRBM33 depletion reduces proliferation and glycolysis, and enhances apoptosis of malignant cells [10]. These reports suggest that circRBM33 is a promoter of cancer progression.

Next, we found that miR-542-3p interacts with circRBM33 and regulates BCa proliferation and progression. For this, we investigated the potential functions of upregulated miR-542-3p in BCa and observed that its overexpression exhibits similar effects to those of circRBM33 downregulation in BCa cells. H-X Wu *et al*. have reported that miR-542-3p is expressed at low levels in BCa, and that the introduction of ectopic miR-542-3p into BCa cells reduces cell growth [14]. Moreover, miR-542-3p mimic-induced upregulation of miR-542-3p in BCa cells strengthens the efficacy of paclitaxel in chemoresistant BCa [11]. On the other hand, a study confirmed that the combination of miR-542-3p expression and, doxorubicin can promote the apoptosis of triple negative BCa cells, indicating a potential use of this combination as a promising therapy for BCa [12]. Similarly, the reduction of miR-542-3p level was tested in colorectal cancer, while its overexpression provides resistance against the aggressive behaviors of cancer cells by negatively modulating the expression of miR-542-3p’s targets [27]. Additionally, another publication reported that restoring miR-542-3p is capable of restraining proliferation, whilst miR-542-3p knockdown exerts the opposite effect on epithelial ovarian cancer cells [28]. In general, all these studies have confirmed our finding that miR-542-3p increased expression depresses tumorigenesis.

Finally, the target of miR-542-3p, HIF-1α, has captured our attention. Through rescue experiment, we noticed that HIF-1α is a miR-542-3p downstream factor in mediating BCa, as shown by the results that demonstrated that HIF-1α overexpression mitigates the role of upregulated miR-542-3p in BCa cells. Recently, it was reported that HIF-1 stabilization has anti-tumor effects on BCa [29]. A former study has showed that Parkin-mediated ubiquitination and degradation of HIF-1α is partly involved in reducing metastasis of BCa cells [30]. HIF-1 activation is an active driver of BCa glycolysis [31], and a marker of enhanced aerobic glycolysis associated with drug resistance [32]. A recent study reported that in cardamonin-treated BCa, HIF-1α inhibition is partly involved in reducing glucose uptake and lactic acid production [17].

## Conclusion

Overall, this study demonstrates that the inhibition of circRBM33 prevents BCa progression by reducing glycolysis and proliferation and by promoting apoptosis of BCa cells. It also shows that this mechanism involves miR-542-3p-mediated downregulation of HIF-1α. Our experimental discovery may provide new insights into the molecular mechanism of BCa. In the future, more studies are required to further validate and develop the results of our research.

## Supplementary information

cddis-author-contribution-form.pdf

Editorial certificate

Supplementary Figure 1

Supplementary Figure 1

aj-checklist.
