# Supplementary material for: CircRBM33 downregulation inhibits hypoxia-induced glycolysis and promotes apoptosis of breast cancer cells via a microRNA-542-3p/HIF-1α axis
Source: Cell Death Discov. 2022 Mar 22;8:126. doi: 10.1038/s41420-022-00860-6 (PMC8941146; doi:10.1038/s41420-022-00860-6)
Supplement: Supplementary file 1 — cddis-author-contribution-form.pdf [file 41420_2022_860_MOESM1_ESM.pdf]

DECLARATION OF CONTRIBUTIONS TO ARTICLE

ADMC

Manuscript Number: CDDISCOVERY-21-2253R3

Journal Name: *Cell Death & Disease* (the 'Journal')

Proposed Title of the Contribution: Authors' contributions (the 'Contribution)

Author(s): Yiming Jiang, Meiqi Zhang, Danlu Yu, Guoxin Hou, Jingyi Wu, Fuming Li (the 'Authors')

For all *CDDis* articles, each person named as an author in the published version must be able to show he or she has contributed substantially to the article.

Authorship credit should be based on 1) substantial contributions to conception and design, acquisition of data, or analysis and interpretation of data; 2) drafting the article or revising it critically for important intellectual content; and 3) final approval of the version to be published. Authors should meet conditions 1, 2 and 3.

Any person who cannot be shown to have made a substantial contribution to the article cannot be listed as an author in the final version. The name of any person who is deemed to have made a minor contribution can, however, appear in the Acknowledgments section of the article.

Please complete the table below to indicate the contributions of all named authors to the manuscript.

Author Full Name:	Specification of Contribution to the Manuscript:
Yiming Jiang, Meiqi Zhang, Danlu Yu, Guoxin Hou, Jingyi Wu, Fuming Li	data analysis
Yiming Jiang, Meiqi Zhang, Danlu Yu, Guoxin Hou, Jingyi Wu, Fuming Li	study design
Yiming Jiang, Meiqi Zhang, Danlu Yu, Guoxin Hou, Jingyi Wu, Fuming Li	experimental studies
Yiming Jiang, Meiqi Zhang	manuscript editing

Please complete the table below to indicate the contributions of all named authors to the figures.

Figure 1:

Yiming Jiang, Meiqi Zhang, Danlu Yu, Guoxin Hou, Jingyi Wu, Fuming Li

Figure 2:

Yiming Jiang, Meiqi Zhang, Danlu Yu, Guoxin Hou, Jingyi Wu, Fuming Li

Figure 3:

Yiming Jiang, Meiqi Zhang, Danlu Yu, Guoxin Hou, Jingyi Wu, Fuming Li

Figure 4:

Yiming Jiang, Meiqi Zhang, Danlu Yu, Guoxin Hou, Jingyi Wu, Fuming Li

Figure 5:

Yiming Jiang, Meiqi Zhang, Danlu Yu, Guoxin Hou, Jingyi Wu, Fuming Li

Figure 6:

Yiming Jiang, Meiqi Zhang, Danlu Yu, Guoxin Hou, Jingyi Wu, Fuming Li

Figure 7, supplementary Figure 1:
Yiming Jiang, Meiqi Zhang, Danlu Yu, Guoxin Hou, Jingyi Wu, Fuming Li

Signed for and on behalf of the Author(s):	Print Name:	Date:
Fuming Li	Fuming Li	20210106
