# Supplementary material for: CircRBM33 downregulation inhibits hypoxia-induced glycolysis and promotes apoptosis of breast cancer cells via a microRNA-542-3p/HIF-1α axis
Source: Cell Death Discov. 2022 Mar 22;8:126. doi: 10.1038/s41420-022-00860-6 (PMC8941146; doi:10.1038/s41420-022-00860-6)
Supplement: Supplementary file 2 — Editorial certificate [file 41420_2022_860_MOESM2_ESM.pdf]

EDITSPRINGS

# EDITORIAL CERTIFICATE

**This is to certify that the manuscript detailed below was edited by one or more of our highly qualified, native English speakers at EditSprings, to assure compliance with Anglophone academic standards in terms of style, punctuation, grammar, and spelling.**

## Manuscript title:

**CircRBM33 downregulation inhibits hypoxia-induced glycolysis and promotes apoptosis of breast cancer cells via a microRNA-542-3p/HIF-1 axis**

## Authors:

**Yiming Jiang, Meiqi Zhang, Danlu Yu, Guoxin Hou, Jingyi Wu, Fuming Li**

## Date Issued:

**Dec 23 2021**

## Certificate Number:

**ES-202112161715912405**

This certificate can be verified on https://www.editsprings.cn/QueryCertificate.html EditSprings hereby certifies that neither content nor the author's intentions were altered in any way during the editing process. Documents in receipt of this certification should be ready for publication as far as style and language are concerned, provided that the author(s) accepted our suggestions and changes (which remains their right and responsibility).

EditSprings offers a wide range of editing, translation, for researchers and publishers across the world. Our highly skilled editors are all established academics based in Anglophone Higher Education institutions across the world (U.K., U.S.A., Canada, Australia, and elsewhere), are experts in their respective fields, and are qualified to edit research papers authored by non-Anglophone scholars.
