# Supplementary material for: CircRBM33 downregulation inhibits hypoxia-induced glycolysis and promotes apoptosis of breast cancer cells via a microRNA-542-3p/HIF-1α axis
Source: Cell Death Discov. 2022 Mar 22;8:126. doi: 10.1038/s41420-022-00860-6 (PMC8941146; doi:10.1038/s41420-022-00860-6)
Supplement: Supplementary file 3 — Supplementary Figure 1 [file 41420_2022_860_MOESM3_ESM.docx]

**Supplementary Figure 1** The effect of up-regulation or down-regulation of miR-149 or miR-758-3p on the expression of HIF-1α. A. The effect of up-regulation or down-regulation of miR-149 on the expression of HIF-1α; B. The effect of up-regulation or down-regulation of miR-758-3p on the expression of HIF-1α.
