# Supplementary figures and images for: CircRBM33 downregulation inhibits hypoxia-induced glycolysis and promotes apoptosis of breast cancer cells via a microRNA-542-3p/HIF-1α axis
Source: Cell Death Discov. 2022 Mar 22;8:126. doi: 10.1038/s41420-022-00860-6 (PMC8941146; doi:10.1038/s41420-022-00860-6)

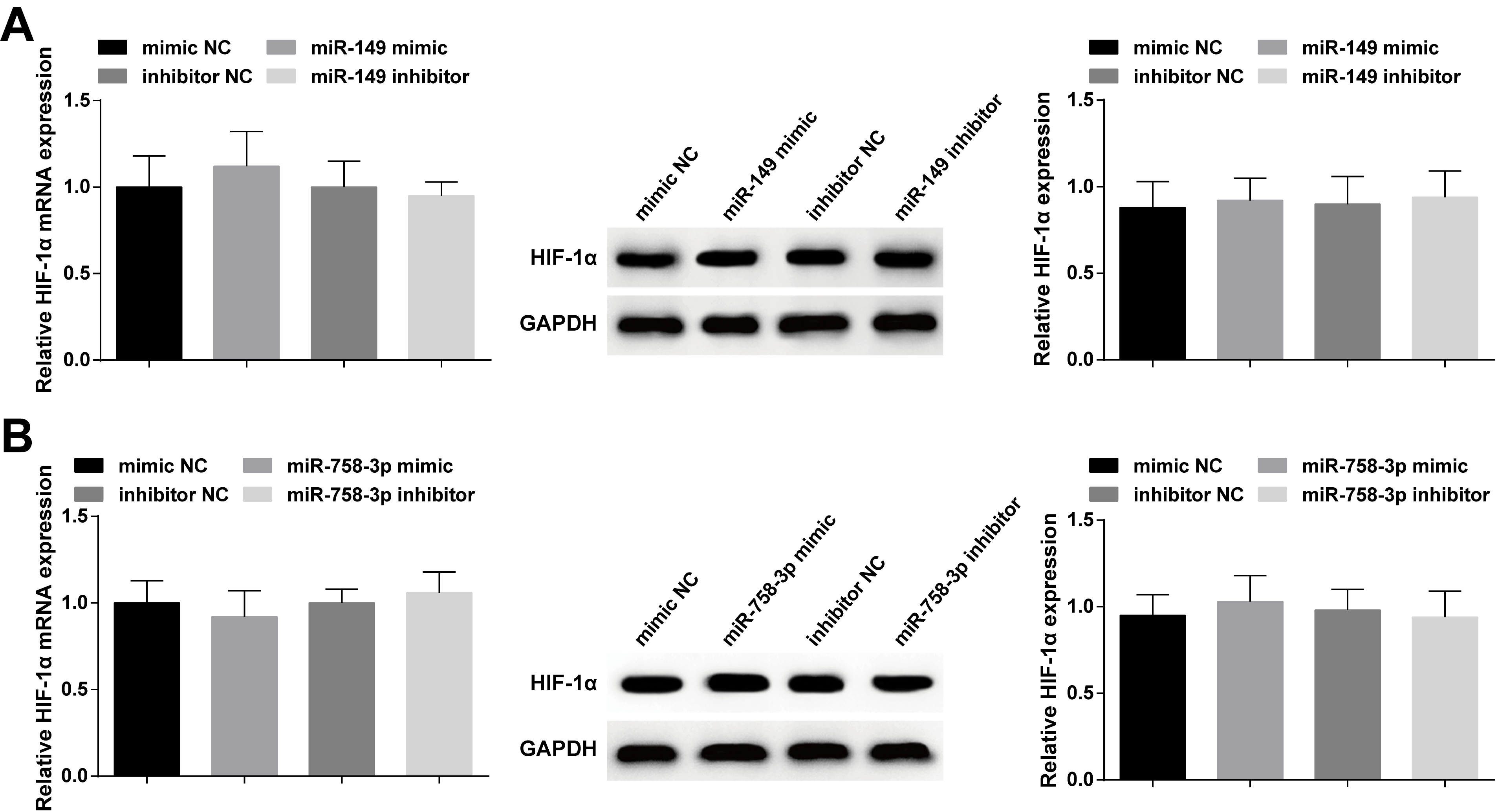

Supplement: Supplementary file 4 — Supplementary Figure 1 [file 41420_2022_860_MOESM4_ESM.tif]
